# Genome-Wide Analysis of Soybean Apyrase Gene Family and Functional Characterization of GmAPY1-4 Responses to Aluminum Stress

**DOI:** 10.3390/ijms26051919

**Published:** 2025-02-23

**Authors:** Yanyu Yu, Shengnan Ma, Lanxin Li, Zhen Song, Lin Yu, Chunshuang Tang, Chunyan Liu, Qingshan Chen, Dawei Xin, Jinhui Wang

**Affiliations:** 1 National Key Laboratory of Smart Farm Technology and System, Key Laboratory of Soybean Biology in Chinese Ministry of Education, College of Agriculture, Northeast Agricultural University, Harbin 150030, China; 15836715092@163.com (Y.Y.); lilanxin0530@163.com (L.L.); cyliucn@126.com (C.L.); qshchen@126.com (Q.C.); 2 Crop Development Research Institute, Heilongjiang Academy of Land Reclamation Sciences, Harbin 150038, China; mashengnan34@163.com (S.M.); nkyulin@sina.com (L.Y.); 13644546046@163.com (C.T.); 3 College of Life Sciences, Northeast Agricultural University, Harbin 150030, China; 18346081762@163.com

**Keywords:** apyrase gene family, soybean, conserved motif, protein structure, cis-regulatory elements, expression pattern, aluminum stress

## Abstract

Apyrases (APYs) directly regulate intra- and extra-cellular ATP homeostasis and play a key role in the process of plants adapting to various stresses. In this study, we identified and characterized soybean APY (GmAPY) family members at the genomic level. The results identified a total of 18 APYRASE homologous genes with conserved ACR domains. We conducted a bioinformatics analysis of *GmAPYs*, including sequence alignment, phylogenetic relationships, and conserved motifs. According to the phylogenetic and structural characteristics, *GmAPYs* in soybeans are mainly divided into three groups. The characteristics of these *GmAPYs* were systematically evaluated, including their collinearity, gene structure, protein motifs, cis-regulatory elements, tissue expression patterns, and responses to aluminum stress. A preliminary analysis of the function of *GmAPY1-4* was also conducted. The results showed that GmAPY1-4 was localized in the nucleus, presenting relatively high levels in roots and root nodules and demonstrating high sensitivity and positive responses under aluminum stress circumstances. Further functional characterization revealed that the overexpression of GmAPY1-4 in hairy roots not only induced root growth under normal growth conditions but also significantly prevented root growth inhibition under aluminum stress conditions and contributed to maintaining a relatively higher fresh root weight. By contrast, RNAi interference with the expression of GmAPY1-4 in hairy roots inhibited root growth under both normal and aluminum stress conditions, but it exerted no significant influence on the dry or fresh root weight. To sum up, these findings support the significant functional role of GmAPY1-4 in root growth and the aluminum stress response. These findings not only enhance our comprehension of the aluminum stress response mechanism by identifying and characterizing the APY gene family in the soybean genome but also provide a potential candidate gene for improving aluminum tolerance in soybeans in the future.

## 1. Introduction

Apyrases (APYs) are a class of nucleoside triphosphate diphosphate hydrolases (NTPDases), which can hydrolyze extracellular ATP (eATP) to AMP and inorganic phosphate [1,2]. Apyrase belongs to the GDA1-CD39 (guanosine diphosphatase 1—cluster of differentiation 39) phosphatase superfamily [3]. The cellular ATP level is strictly controlled by the GDA1-CD39 nucleoside phosphatase family [3]. Expressed by prokaryotic cells and most eukaryotic cells, apyrase was first identified in plants through its extraction from potato tubers [4,5]. Since then, it has been extensively studied in various plants, including *Arabidopsis thaliana* [6], wheat [7], rice [8], *Medicago truncatula* [9], *Phaseolus vulgaris* [10], potatoes [11], *Mimosa pudica* [12], and *Populus euphratica* [13]. It was found that apyrase plays an important role in plant growth and development and stress responses.

APYs have an impact on the development of plant root systems, pollen germination, and seed yields. AtAPY1 and AtAPY2 are precisely localized within the Golgi apparatus and participate in the regulation of cell elongation, root growth, and pollen tube germination [1,14,15]. The double knockout mutant of *apy1* and *apy2* manifests a dwarfing phenotype and significantly restricts the growth of primary roots by inhibiting the elongation of root cells [16]. AtAPY7 serves as a regulatory factor and constitutes a component of the LRX/RALF/FER signaling module, being involved in the coordination of cell wall construction and cell proliferation [17]. In the case of constitutive expression of the pea *psNTP9* gene, it results in the expansion of root systems of soybean plants, enhancement of their drought resistance, and a marked increase in seed yields [18].

APYs assume a vital role in the response to adverse circumstances. The apyrase of the *Arabidopsis* family is a protein group with critical biological functions comprising seven members from AtAPY1 to AtAPY7. AtAPY1 mediates a response to low boron availability by controlling cell wall integrity under boron deficiency conditions [19]. Although AtAPY1 and AtAPY2 are endo-APYs, their mutation could cause significant elevation of extracellular ATP (eATP) [20]. The transcription levels of AtAPY1 and AtAPY2 of *Arabidopsis thaliana* seedlings were significantly increased under hypertonic stress in order to increase the eATP concentration for survival [16,21]. At the same time, transcription levels of NADPH oxidase, AtMAPK3, and AtACS6 increased in the eATP signaling-induced antioxidant system and MAPK immune response to stress mechanisms [16]. Microarray and quantitative real-time PCR analyses have demonstrated that AtAPY1, AtAPY2, and eATP play crucial roles in linking biotic stress to plant defense responses and growth adaptation [22]. The overexpression of PeAPY2 potentiates the cold tolerance of plants through modulating vesicle trafficking and extracellular ATP levels [23]. Overexpression of *Populus euphratica PeAPY1* and *PeAPY2* reduces the stomatal density, decreases leaf water loss rates, and enhances the water retention capacity [24]. Therefore, stress-induced APYs play a core regulatory role in the process of plants adapting to environmental stress.

In light of the key role of APY genes in the response to abiotic stress, the identification and functional characterization of APY family genes can offer new potential targets for the improvement of crop stress resistance. At present, research related to APY genes in soybean remains relatively scarce. In this research, we identified the APY members in soybean at the genomic level by employing bioinformatics tools and carried out a comprehensive phylogenetic analysis of them via bioinformatics.

Soybean is one of the most significant food and oil crops globally [25,26]. Soil acidification, induced by the emission of acidic gases and the excessive application of nitrogenous fertilizers, results in the aggravation of heavy metal stress [27]. In the case of aluminum toxicity stress especially, it affects the growth of soybeans and poses a threat to the yield of soybeans. In the face of aluminum toxicity stress in soil, the cellular structure of soybean root systems is impaired; the eATP levels both inside and outside of the cells are imbalanced, resulting in the inhibition of soybean root growth [28]. APYs are capable of regulating the homeostasis of ATP both intracellularly and extracellularly and play a crucial role in the process of plant root development. Consequently, this research primarily investigated the response mechanism of GmAPYs under aluminum stress.

Investigating the function of the soybean Apyrases family under aluminum stress not only contributes to uncovering the molecular mechanism of plant aluminum tolerance but also offers theoretical grounds and technical support for cultivating aluminum-tolerant crop varieties. This study holds significant importance for enhancing crop yields in acidic soils and facilitating sustainable agricultural development.

## 2. Results

### 2.1. Identification of the GmAPY Family

Using the TAIR database, seven members of the *AtAPY* family were identified: *AT3G04080* (*AtAPY1*), *AT5G18280* (*AtAPY2*), *AT1G14240* (*AtAPY3*), *AT1G14230* (*AtAPY4*), *AT1G14250* (*AtAPY5*), *AT2G02970* (*AtAPY6*), and *AT4G19180* (*AtAPY7*). Subsequent BLAST (https://phytozome-next.jgi.doe.gov/blast-search, accessed on 5 October 2024) analyses identified 18 *GmAPY* genes with the conserved ACR domain in soybean (Figure S1).

The proteins encoded by GmAPY genes exhibited remarkable diversity in terms of length, isoelectric point, and molecular weight. The proteins encoded by these *GmAPY* genes were found to vary significantly in length, ranging from 255 to 730 amino acids. With respect to their protein characteristics, the isoelectric points of the proteins encoded by these *GmAPYs* ranged from 5.04 to 9.45, while their relative molecular weights ranged from 27.73 kDa to 81.97 kDa (Table S1).

Subcellular localization predictions revealed that most *GmAPY*-encoded proteins were likely to localize to the nucleus, with the exception of *Glyma.05G039650*, which was predicted to localize to the cell membrane (Table S2). *Glyma.05G039650* encodes an alkaline protein composed of 483 amino acids, and its theoretical isoelectric point is 8.79. The structure of this protein was predicted using AlphaFold, and it was found that its N-terminal encompasses a typical α-helical transmembrane domain. This structural characteristic may account for the localization of this protein on the cell membrane (Figure S2).

### 2.2. Analyses of GmAPY Homology and Gene Structures

**Figure 1:**
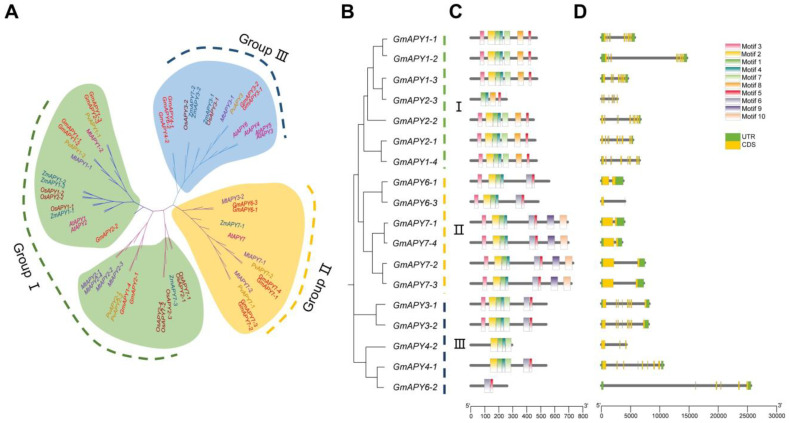
Phylogenetic, conserved motif, and gene structure analyses of the soybean APY gene family. (**A**) A phylogenetic tree of the APY proteins encoded by *G. max*, *A. thaliana*, *Z. mays*, *O. sativa*, *P. vulgaris*, and *M. truncatula*. Groups I, II, and III are shown in light green, light yellow, and light blue, respectively. (**B**) A phylogenetic tree incorporating the identified GmAPY proteins. (**C**) The distributions of motifs 1–10 (represented with differently colored rectangular boxes) in GmAPY proteins. (**D**) GmAPY gene structures arranged based on their phylogenetic relationships. Shown with green boxes are 5′ UTR and 3′ UTR, while exons are denoted with yellow boxes, and introns are represented by gray lines.

Comparative analyses of soybean APYs with homologs in *Medicago truncatula*, *Phaseolus vulgaris*, *Arabidopsis thaliana*, *Oryza sativa*, and *Zea mays* were used to elucidate their phylogenetic relationships. Based on these analyses, *GmAPYs* were named according to their positions relative to *APY* homologs (Table S3). The evolutionary tree clustering analysis showed that *GmAPYs* were categorized into three distinct groups (Figure 1A). Specifically, in soybeans, Group I contained seven members, Group II had six members, and Group III consisted of five members (Figure 1B).

The MEME program was employed to analyze the conserved motifs of the GmAPYs. The analysis identified 10 conserved motifs (motifs 1–10) (Table S4). All GmAPY family members encompassed at least two or more conserved motifs. The members within each phylogenetic group manifested similar motif distribution patterns.

Specifically, in Group I of the phylogenetic tree (Figure 1C), all members shared the identical motif distribution pattern, with the majority comprising seven motifs: 1, 2, 3, 4, 5, 7, and 8. Notably, Motif 8 was a distinctive feature of this group, yet GmAPY2-3 was an exception, merely containing motifs 1, 4, 5, and 8.

In Group II, the motif distribution was largely consistent, with most members containing motifs 1, 2, 3, 4, 5, 6, 9, and 10. However, GmAPY6-1 and GmAPY6-3 lacked motifs 9 and 10, which were otherwise unique to this group. Group III exhibited certain variances. Although the majority of the members contained motifs 1, 2, 4, and 7, GmAPY6-2 was an exception, retaining only motifs 5 and 6. The aforementioned results not only disclosed the evolutionary conservation of common motifs among groups but also accentuated the unique motif patterns within each group, suggesting that APYs might have functional differentiations in different phylogenetic groups.

To further investigate the genomic organization associated with the apyrase family, the exon and intron structures of *GmAPYs* were studied next. The number of exons in the *GmAPYs* varied significantly, ranging from 1 to 9. Group I’s *GmAPYs* exhibited a high degree of conservation, with all members consistently containing nine exons. Group II typically contains two exons, but *GmAPY6-3* had only one exon, which was the fewest among all of the *GmAPYs*. In contrast, Group III showed greater variability, with the number of exons ranging from a minimum of 3 to a maximum of 8, as well as some having 5 exons. Although the exon lengths of members within the same group were relatively consistent, there were significant differences in the intron lengths (Figure 1D). This pattern suggests that phylogenetically related members share similar gene structures, reflecting evolutionary principles.

### 2.3. Analyses of GmAPY Synteny

**Figure 2:**
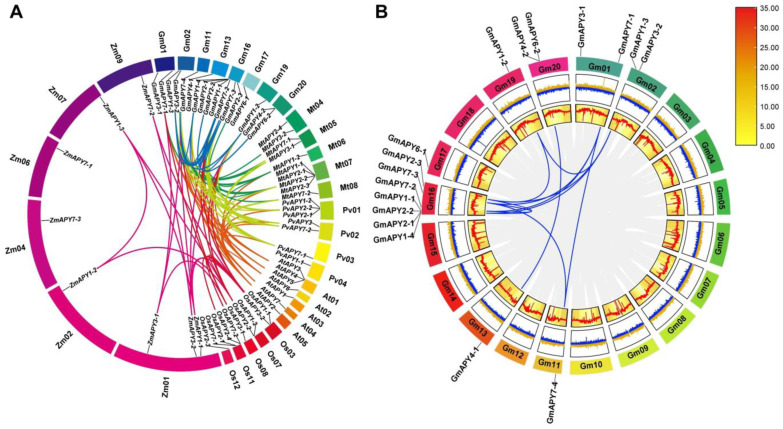
Chromosomal localization, synteny, and homology analyses of the *GmAPY* gene family. (**A**) Homology between *GmAPYs* and corresponding *APY* genes in *A. thaliana*, *Z. mays*, *O. sativa*, *P. vulgaris*, and *M. truncatula* was assessed. Chromosomes for these species are represented using circles, while syntenic APY regions are marked with colored curves. (**B**) Analyses of the internal synteny within the *GmAPY* family were conducted. Blue lines represent duplicated gene pairs, with coloration being proportional to log2 expression, and yellow and red indicate low and high density levels, respectively. Chromosomes are represented using colored boxes, while genomic blocks with orthologous relationships in the soybean genome are marked using gray lines, and homologous *GmAPY* gene pairs are outlined with blue lines.

To investigate duplication events within the *GmAPY* gene family, a collinearity analysis was next conducted with MCScanX in Tbtools-II (v2.096). This analysis revealed that all *GmAPY* genes originated from segmental duplication events. A collinearity analysis between *GmAPY* genes and *APY* genes encoded by five other species (*P. vulgaris*, *M. truncatula*, *A. thaliana*, *O. sativa*, and *Z. mays*) identified 15 pairs, 13 pairs, 11 pairs, 6 pairs, and 5 pairs of orthologous gene pairs, respectively (Figure 2A, Table S5). This indicates that GmAPYs have a closer evolutionary relationship with the two leguminous plant apyrases (PvAPYs and MtAPYs), which is consistent with the results of the sequence similarity analysis.

Further investigation of these collinear genes revealed that some *GmAPYs*, such as *GmAPY1-2* and *GmAPY3-2*, were involved in multiple collinear gene pairs across these five species. Specifically, nine *GmAPYs* (*GmAPY1-2*, *GmAPY1-3*, *GmAPY1-4*, *GmAPY2-3*, *GmAPY3-2*, *GmAPY7-1*, *GmAPY7-2*, *GmAPY7-3*, and *GmAPY7-4*) showed collinearity with *P. vulgaris*, while four were collinear with *O. sativa* (Figure 2A, Table S5). The number of collinear genes was greater with dicotyledonous plants than with monocotyledonous plants, further supporting the phylogenetic relationships inferred from the above analyses. Moreover, 11 paralogous APY gene pairs were discovered in the soybean genome. These duplicate genes often occur in related species (Figure 2B, Table S6). These synteny occurrences proved that many APY genes had already evolved before the divergence of soybean species.

### 2.4. Cis-Acting Element Analyses of GmAPYs

**Figure 3:**
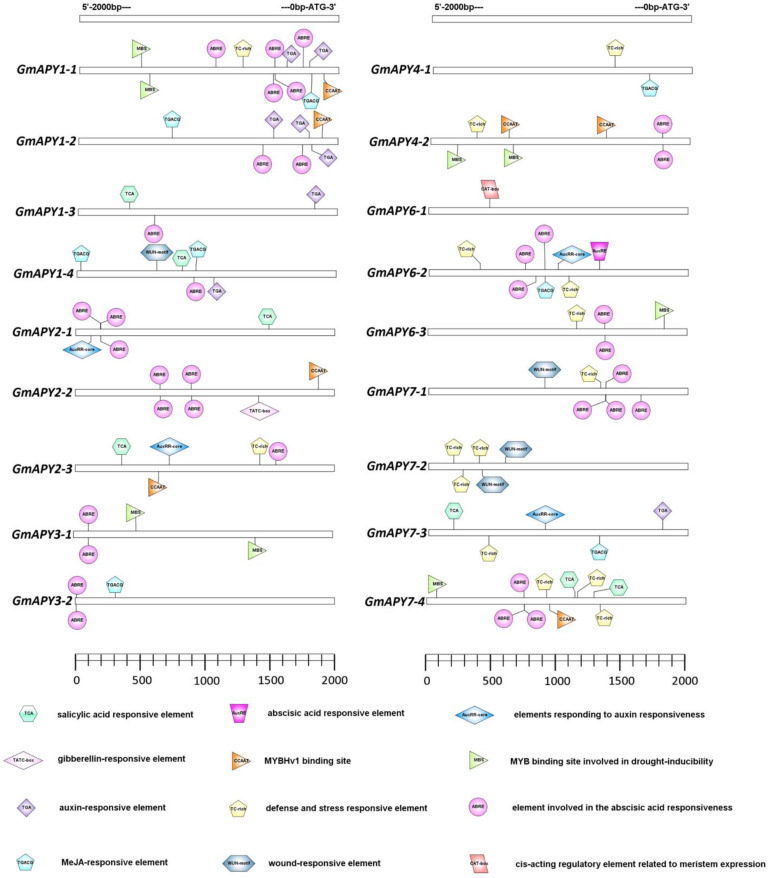
Predictive analyses of the cis-acting elements present in the promoters upstream of *GmAPYs*. Different colored boxes denote the relative positions of cis-acting elements associated with each of these *GmAPYs*.

Using the PlantCARE database, in-depth analyses of the cis-regulatory elements located within the 2000 bp promoter regions upstream of these *GmAPYs* were then conducted to investigate their potential roles in transcriptional regulation (Figure 3). Stress- and hormone-related regulatory elements were identified in all three groups established above.

In Group I, stress-related elements such as CCAAT, MBS, and TC-rich repeats, which are likely involved in plant responses to environmental stress, were identified. Additionally, hormone-related elements, including TGA, ABRE, TCA, AuxRR-core, and TGACG motifs, were observed, suggesting their involvement in hormone signaling pathways. Group II members of this family were found to harbor growth- and development-related CAT elements, alongside stress-related elements like WUN-motif, CCAAT, MBS, and TC-rich repeats, as well as hormone-related motifs such as ABRE and TGACG. In Group III, stress-related elements like CCAAT, MBS, and TC-rich repeats were again prevalent, alongside hormone-related elements like ABRE, TGACG, and AuxRR-core motifs.

The presence of these cis-acting elements in the promoter regions of most GmAPYs suggests that this gene family plays a vital role in environmental stress responses and hormone signaling pathways [29].

### 2.5. Characterization of Tissue-Specific GmAPY Expression in Different Tissues and Under Aluminum Toxicity Stress

**Figure 4:**
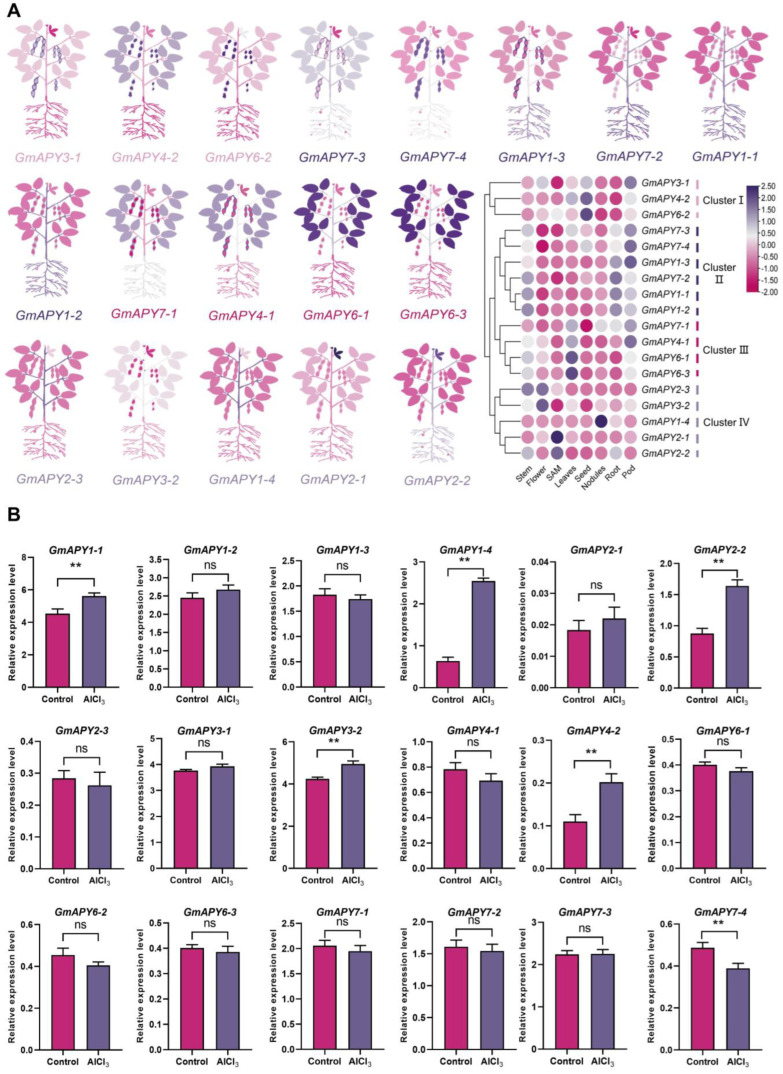
Analyses of the expression of *GmAPYs* in different tissues and in response to AlCl_3_ stress. (**A**) *GmAPY* expression levels were analyzed across soybean tissue types with the Phytozome database, with a heatmap having been constructed with TBtools showing the log2 expression levels. Pink and purple denote low- and high-abundance transcripts, respectively. (**B**) *GmAPY* expression levels under conditions of AlCl_3_ stress. Three biological replicates were established for all experiments. ** *p* < 0.01 (*t*-test). Data are expressed as means ± SEM (*n* ≥ 3).

Gene expression patterns provide valuable insights into the potential biological functions of genes. Subsequently, we conducted a systematic analysis of the expression profiles of 18 *GmAPYs* in different tissues of soybean, including flowers, leaves, nodules, pods, roots, seeds, SAM, and stems. The results showed that the *GmAPY* transcripts were expressed in all tissues, but their expression levels varied significantly. Based on these expression levels, we classified *GmAPYs* into four distinct expression pattern clusters (Figure 4A, Table S7). Cluster 1’s genes showed high expression in the seeds and pods but low expression in the roots and nodules. Cluster 2’s genes were actively expressed in the stems, roots, and pods but had low expression in the flowers and SAM. Cluster 3’s genes were highly expressed in the leaves and pods but had lower expression in the seeds and SAM. Cluster 4’s genes exhibited varied expression patterns. For instance, *GmAPY1-4* was mainly highly expressed in the root nodules, while *GmAPY2-1* and *GmAPY2-2* had higher expression in the shoot tips, and *GmAPY7-1* had the lowest expression in the seeds. These different expression patterns suggest that *GmAPYs* play multiple roles in the growth and development of soybean.

Furthermore, under the aluminum stress condition of 50 μmol L^−1^ AlCl_3_, we evaluated the expression patterns of the *GmAPY* genes to explore their roles in the non-biotic stress response. The results showed that most of the *GmAPY* genes responded to aluminum stress, among which *GmAPY1-1*, *GmAPY1-4*, *GmAPY2-2*, *GmAPY3-2*, and *GmAPY4-2* were significantly upregulated. Notably, the expression of GmAPY1-4 increased approximately threefold under aluminum stress. In contrast, *GmAPY7-4* was significantly downregulated under the same condition (Figure 4B). These results suggest that *GmAPYs*, especially GmAPY1-4, play important roles in the aluminum-induced stress response, highlighting their potential functions in non-biotic stress adaptation.

### 2.6. Subcellular Localization Analyses of GmAPY1-4

**Figure 5:**
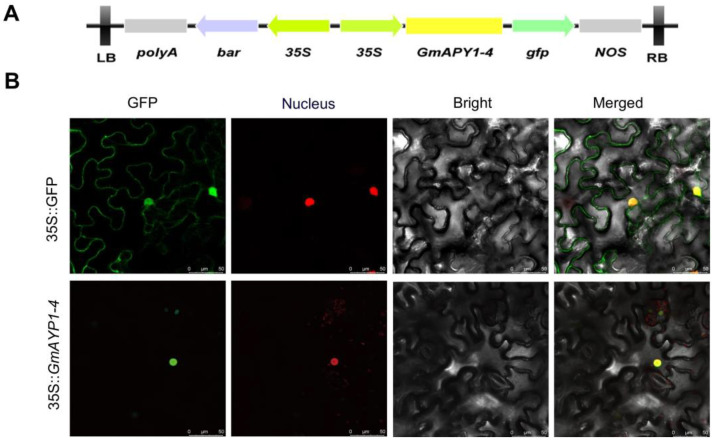
Subcellular localization analysis of GmAPY1-4. (**A**) Schematic overview of the utilized pSOY1-GmAPY1-4 vector, with expression elements marked with arrows. (**B**) Subcellular localization analyses of GmAPY1-4 were conducted in tobacco leaves, with confocal images showing the localization of GFP and GmAPY1-4-GFP. Scale bar = 50 μm.

Given that GmAPY1-4 showed a significant response under aluminum stress conditions, this gene was selected as the key object for further experimental investigation. To determine the subcellular localization of the protein encoded by *GmAPY1-4*, a GFP fusion protein expression system was constructed (Figure 5A, Table S9). The constructed expression vector was used to transform tobacco cells. The situation three days after transformation was observed under a confocal microscope (Figure 5B). In the control group containing the empty vector, the GFP signal was uniformly distributed throughout the cells, while in the experimental group expressing the *GmAPY1-4*-GFP fusion protein, the GFP signal was specifically localized to the nucleus. These findings align with the subcellular localization prediction by the Cell-PLoc software (http://www.csbio.sjtu.edu.cn/bioinf/Cell-PLoc-2/, accessed on 26 October 2024).

### 2.7. GmAPY1-4 Regulates Transgenic Soybean Hairy Root Phenotypes in Response to Aluminum Stress

**Figure 6:**
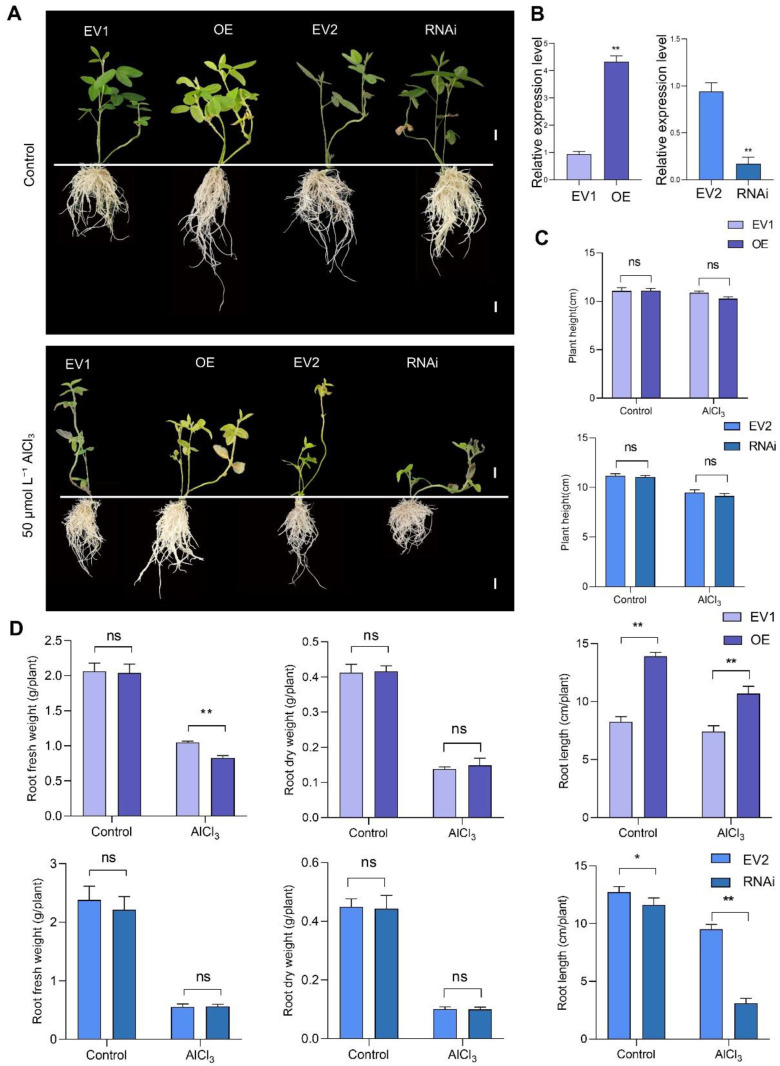
Transgenic GmAPY1-4 hairy roots’ altered root architecture. (**A**) The phenotypes of soybean hairy roots overexpressing GmAPY1-4 (OE), its control EV1, RNAi GmAPY1-4 soybean hairy roots (RNAi), and its control EV2 under normal growth conditions and after 7 days of 50 µM AlCl_3_ stress. Scale bar = 1 cm. (**B**) The expression of GmAPY1-4 in the roots of EV1, OE, EV2, and RNAi plants was analyzed via real-time quantitative reverse transcription polymerase chain reaction (qRT-PCR). (**C**) The changes in plant height for the EV1, OE, EV2 and RNAi plants under normal growth conditions and after 7 days of 50 µM AlCl_3_ stress. (**D**) Under normal growth conditions and after 7 days of treatment with 50 µM AlCl_3_ stress, the fresh weight of the roots, dry weight of the roots, and maximum root length of the EV1, OE, EV2 and RNAi plants. Statistical significance: * *p* < 0.05; ** *p* < 0.01 (Student’s *t*-test). Data are presented as the mean ± standard error (*n* ≥ 3).

To further elucidate the functions of *GmAPY1-4*, this research established the RNA interference (RNAi) and overexpression (OE) systems of GmAPY1-4 in soybean hairy roots. The successful overexpression and RNA interference of *GmAPY1-4* were confirmed using quantitative real-time PCR (qRT-PCR) (Figure 6B). Under normal growth conditions, the root length of overexpression (OE) was significantly longer than that of the empty vector control (EV1), although there were no significant differences in root dry weight or root fresh weight. Under conditions of aluminum stress, the OE displayed significantly longer root lengths and higher root fresh weights compared with EV1, with no notable differences in root dry weight (Figure 6A,C,D).

Similarly, under normal growth conditions, significant phenotypic differences were observed between the RNAi lines and the empty vector control (EV2). The root length of the RNAi lines was shorter than that of EV2, but no significant differences were observed in the root dry weight or fresh weight. Under aluminum stress, the RNAi exhibited severe leaf wilting and significantly shorter root lengths compared with EV2, but again, no significant differences were noted in the root dry weight or fresh weight (Figure 6A,C,D).

These analyses revealed that under both normal and aluminum stress conditions, overexpression of *GmAPY1-4* was able to promote root growth (as indicated by increased root length) while enhancing the root fresh weight under aluminum stress. In contrast, RNAi-mediated silencing of *GmAPY1-4* inhibited root growth (as evidenced by reduced the root length) without affecting the root dry or fresh weight under either condition. These findings suggest that *GmAPY1-4* plays a critical role in regulating root growth and plant responses to aluminum stress.

## 3. Discussion

APYs are capable of maintaining the homeostasis of ATP both intracellularly and extracellularly, and they play a crucial role in the regulation of various stress adaptations in plants [30,31,32]. Aluminum stress can result in the accumulation of a considerable amount of extracellular eATP, thereby triggering an increase in reactive oxygen species (ROS) levels and eventually inducing cell apoptosis [31,33]. Recent studies have demonstrated that overexpression of the APY gene can markedly inhibit the generation of ROS, confirming that APYs might be an effective target for enhancing plant stress resistance [23]. With the precise sequencing and assembly of the soybean genome, the identification of GmAPY family members has been made feasible, providing a foundation for studying their functions. In this research, we performed comprehensive identification and characterization of GmAPY family members at the genomic level. The results show that a total of 18 *APY* genes, all containing conserved ACR domains, were identified in the soybean genome.

Seven APY members were identified in *Arabidopsis thaliana*, 27 were identified in wheat [7], and 17 were identified in peanuts (*Arachis hypogaea*) [34]. These members were classified into three disparate groups in accordance with their evolutionary affinities. To better analyze the structure and function of the *GmAPYs*, a phylogenetic tree encompassing APY genes from soybean, *A. thaliana*, *M. truncatula* [35], *P. vulgaris*, *Z. mays* [36], and *O. sativa* [8] was constructed. According to the phylogenetic relationship, we divided the *GmAPY* gene family into three groups. This result is consistent with the AtAPY gene family and AhAPY gene family divided into three groups [8]. Although the APY family has undergone remarkable expansion during the course of evolution, its evolutionary mode remains conservative. There exists a close genetic relationship between soybean APY (GmAPY) and its homologous genes in leguminous plants such as PvAPY and MtAPY (*P. vulgaris* and *M. truncatula*), which further substantiates the conservatism of the evolution of the APY family (Figure 1A).

Conserved motifs serve as significant indicators for protein function, structure, and evolution [37]. Within the GmAPYs family of soybeans, Motif 1 and Motif 4 play a crucial role in the catalytic process of apyrases. However, there exist notable disparities in the distribution and functional status of these motifs among different members (Figure 1C, Figure S3, Table S8). Specifically, Motif 1 functions as a proton acceptor, facilitating the activation of water molecules by accepting protons and thereby driving the catalytic reaction and potentially participating in the activation of substrates or the stabilization of reaction intermediates. Motif 4, conversely, acts as an ATP binding site, being responsible for recognizing and binding ATP or ADP. It is directly involved in the hydrolysis of ATP and ADP and constitutes the core region for catalytic function. GmAPY6-2 is deficient in motifs 1 and 4, and thus it may not be engaged in the catalytic process but rather exert other functions. GmAPY2-3 possesses only functional Motif 1 and might only be involved in proton transfer and unable to accomplish ATP/ADP hydrolysis. GmAPY4-1 and GmAPY4-2 have only functional Motif 4 and might act as ATP and ADP sensors or regulatory proteins. The absence or retention of these motifs in different members reflects the functional differentiation and diversity of the GmAPY family.

Gene evolution and expression in plants are heavily influenced by the chromosomal gene location [38]. *GmAPY* genes were found to be unevenly distributed across chromosomes 1, 2, 11, 13, 16, 17, 19, and 20, with the highest number located on chromosome 16. Gene structure analysis showed that no *GmAPY* genes were intronless, and variation in the exon-intron number may reflect functional diversification during evolution (Figure 1D). The collinearity analysis revealed evolutionary relationships between the *GmAPY* and APY genes from *A. thaliana*, *M. truncatula*, *P. vulgaris*, *Z. mays*, and *O. sativa*, suggesting that the *GmAPY* gene family predates the divergence of these species (Figure 2A). Existing research on APY genes in *Arabidopsis thaliana* provides a valuable reference for further studies [39].

To investigate the transcriptional regulatory mechanisms and potential functions of the *GmAPY* gene family, an analysis of the cis-acting elements located within the promoter regions 2000 bp upstream of these genes was conducted. This approach ultimately revealed the presence of five hormone-responsive elements [40], including those sensitive to abscisic acid [41], gibberellin [42], auxin [43], salicylic acid [44], and methyl jasmonate [45], suggesting that the *GmAPY* gene family may participate in multiple hormone signaling pathways. Apyrase exerts a crucial role in auxin transportation and the modulation of stomatal aperture. It was discovered in the experiments measuring the hypocotyls and primary roots of *Arabidopsis thaliana* seedlings that interfering with the expression of AtAPY1 and AtAPY2 would suppress the polar transport of auxin [46]. AtAPY1 and AtAPY2 are expressed at a relatively high level in guard cell protoplasts. The extracellular nucleotides and apyrase of *Arabidopsis* regulate stomatal aperture. The expression of both of these apyrases is related to the conditions conducive to stomatal opening. In *Arabidopsis*, eATP and apyrase can regulate the aperture of guard cells [47]. Under drought conditions, ectopic expression of the constitutive gene psNTP9 results in a decreased rate of water loss from leaves and simultaneously causes stomata to be more sensitive to ABA-induced closure, thereby reducing water loss from leaves [18,48]. Additionally, defense and stress response elements were identified upstream of *GmAPYs* in this study, as were cis-acting elements associated with meristem expression. These hormone- and stress-responsive elements are thus likely to play an important role in regulating the expression of *GmAPY* genes, thereby enhancing plant stress resistance.

To gain a comprehensive understanding of GmAPY expression profiles, their expression patterns were analyzed across several different tissue types. *GmAPY* transcripts were detected in flowers, leaves, nodules, pods, roots, seeds, shoot tips, and stems through these analyses, and further comparisons revealed that their expression levels varied significantly between tissues. Based on these findings, the *GmAPY* genes were categorized into four distinct tissue expression patterns. The first pattern exhibited high transcriptional levels in the seeds and low levels in the roots. This may reflect the role of these GmAPY genes as mediators of the hydrolysis of ATP and ADP as a means of supplying energy for seed germination and early development [49], whereas roots, with relatively stable metabolic activity relative to that observed in seeds, require less energy such that they are not as reliant on these enzymes. The second pattern presented with high transcriptional levels in the pods and low levels in the flowers and shoot tips. Notably, the promoter regions of the *GmAPY* genes associated with this pattern contained the auxin-responsive TGA element [50], indicating that their transcriptional activity may be influenced by auxin, ultimately promoting pod development and maturation. This aligns with the findings for *Arabidopsis*, where suppression of apyrase activity has been demonstrated to reduce polar auxin transport while inhibiting the growth of these plants [6]. The third pattern displayed high transcriptional levels in the leaves and low levels in the seeds. Based on the observed findings, it is possible that these *GmAPYs* control stomatal movement, thereby allowing them to shape water transpiration and gas exchange [51]. The less intense metabolic activity which occurs in seeds may contribute to lower overall energy demands such that these enzymes are not expressed at high levels in this compartment. The fourth pattern was characterized by low transcriptional levels in the seeds. Overall, these patterns offer unique and unprecedented insight into the tissue-specific functional specialization of the *GmAPY* genes in soybean plants.

The impact that aluminum stress had on the *GmAPY* expression profiles was also explored in great detail. When these plants were exposed to aversive aluminum stress, a majority of these *GmAPYs* exhibited shifts in their expression profiles, with several exhibiting significant changes in their expression, including *GmAPY1-1*, *GmAPY1-4*, *GmAPY2-2*, *GmAPY3-2*, and *GmAPY4-2*. Strikingly, *GmAPY1-4* expression levels rose roughly threefold compared with the control conditions, suggesting a possible functional role for this gene as an important mediator which helps soybean plants cope with exposure to aluminum-related stress conditions. Conversely, aluminum stress triggered a significant drop in *GmAPY7-4* expression, possibly suggesting its inhibition under these conditions or its involvement in a physiological process which was impaired or subverted following exposure to aluminum. The distinct expression patterns exhibited by *GmAPYs* exposed to aluminum stress were closely associated with the mechanisms through which plants adapt to being exposed to aluminum stress.

At present, the two most commonly used methods for subcellular localization of soybean protein are using *Arabidopsis* protoplasts and *Nicotiana* leaves. We chose *Nicotiana* leaves as the research platform to conduct subcellular localization analysis of GmAPY1-4, and the results showed that GmAPY1-4 was localized in the nuclei of *Nicotiana* leaf cells. Given that *Nicotiana* leaf cells have unique structural features, such as a smaller cytoplasmic proportion and a specific epigenome, this may have affected the protein localization results. Therefore, in subsequent studies, we will explore other methods to further verify the subcellular localization of GmAPY1-4.

When a promoter analysis of *GmAPY1-4* was conducted, this led to the detection of many different hormone-responsive cis-acting elements, including those sensitive to salicylic acid, auxin, abscisic acid, and methyl jasmonate. These elements are key players which help coordinate the ways in which plants respond upon exposure to abiotic stress. The elevated expression of GmAPY1-4 observed under aluminum stress conditions may be attributed to these regulatory elements. Salicylic acid (SA), a critical signaling molecule involved in stress responses, is capable of enhancing aluminum-induced citrate secretion in roots, reducing aluminum accumulation at the root tips, and mitigating aluminum-induced inhibition of the process of root elongation [52]. Similarly, abscisic acid (ABA) is capable of functioning as an aluminum stress signal, with the application of exogenous ABA contributing to the enhancement of aluminum resistance in soybeans by increasing endogenous ABA levels in the root tips [53]. Additionally, ethylene production induced by aluminum stress is likely to serve as a signal by disrupting the auxin polar transport mediated by AUX1 and PIN2 to modify the auxin distribution in roots and consequently cause the cessation of root elongation [54]. Aluminum stress suppresses polar auxin transport and the formation of starch granules, resulting in an elevated root gravitropic set angle (GSA) and reconfiguration of the root system architecture (RSA), thereby modulating the gravitropism of pea (*P. sativum*) roots [55]. Aluminum stress activates NADPH oxidase on the plasma membrane, which catalyzes the generation of superoxide radicals (O_2_^−^) from oxygen [56]. Concurrently, the Fenton reaction is expedited, converting hydrogen peroxide (H_2_O_2_) into highly toxic hydroxyl radicals (OH^−^) [57] and resulting in excessive accumulation of reactive oxygen species (ROS) within the plant, which ultimately induces oxidative stress [58]. Owing to their inherent reactivity, these species can interact with and damage the membranes of cells such that overall cellular function and integrity can readily become compromised [59]. ROS are also capable of dealing direct damage to the DNA such that strand breaks and damage to individual nucleotide bases can arise, culminating in the disruption of appropriate metabolic activity and the consequent impairment of normal plant growth and development. The biological generation of ROS refers to the generation process of ROS within organisms. Within plants, the generation and accumulation of ROS are affected by multiple regulatory factors. Studies have shown that under aluminum stress conditions, auxin and methyl jasmonate are capable of regulating the accumulation of ROS, thereby playing a crucial role in mitigating aluminum-induced plant damage [46,60,61].

Aluminum stress can suppress the growth of soybean roots, leading to reductions in taproot and lateral root elongation lengths [62]. These changes can also coincide with a drop in the root surface area and overall root volume as concentrations of aluminum in the soil rise [63]. As aluminum concentrations become increasingly elevated, this can coincide with diminished root branching and the overall simplification of the structural composition of the root system for soybean plants. Exposure to aluminum stress can also limit soybean root system vitality, impairing the ability of these plants to absorb water and nutrients [64]. As a consequence, the exposed plants experience a deleterious net drop in soybean growth, yield, and quality.

To further investigate the effect of GmAPY1-4 under normal growth conditions and in the aluminum stress environment, the present study established RNA interference (RNAi) and overexpression (OE) systems in soybean hairy roots. Under normal growth conditions, the OE plants exhibited a comparable plant height to that of the empty vector control (EV1) plants (Figure 6C), but the root length of the OE plants was significantly longer than that of the EV1 plants (Figure 6D). This finding demonstrates that the overexpression of GmAPY1-4 exerted a limited influence on the growth of the aboveground parts of the plant, while the overexpression of this gene might have facilitated the growth or development of the root system. While the RNAi plants did not differ in height from the corresponding EV2 control plants (Figure 6C), they conversely exhibited roots which were shorter than those of the EV2 plants, confirming that silencing GmAPY1-4 resulted in the suppression of root growth. Interestingly, neither the OE nor RNAi plants exhibited any significant differences in root dry weight or fresh weight in comparison with their respective EV1 and EV2 controls. This suggests that GmAPY1-4 primarily influences root elongation rather than having any pronounced effect on biomass accumulation. These findings place an emphasis on the pivotal role which GmAPY1-4 plays in the promotion of root growth under normal growth conditions and in the aluminum stress environment.

When these plants were exposed to aluminum stress conditions, the root lengths of the OE plants significantly exceeded those of the EV1 plants, further validating the role of GmAPY1-4 in promoting root growth under aluminum stress. Furthermore, this outcome indicates that GmAPY1-4 holds significant worth as a potential gene for enhancing the aluminum tolerance capacity of roots. Conversely, the root length of the RNAi plants subjected to aluminum stress was conspicuously shorter than that of EV2 plants, suggesting that the inhibition of GmAPY1-4 expression exerted an additional adverse effect on root growth. This finding further emphasizes the critical function of GmAPY1-4 as a significant mediator in root aluminum tolerance.

Additionally, when these plants were exposed to aluminum stress, there was no observable difference in plant height when comparing the OE and EV1 plants (Figure 6C), However, the root fresh weight of the OE plants remained significantly higher than that of the EV1 plants, suggesting that the OE plants are better equipped to maintain the physiological function and water status in their roots under these avid aluminum stress conditions, thereby preserving a higher fresh weight. Even with this apparent increase in fresh weight, no significant difference in root dry weight was detected between the OE and EV1 plants. This indicates that the OE plants primarily sustain root physiological function by retaining water under conditions of aluminum stress rather than by accumulating additional dry matter. Similarly, RNAi plants and EV2 plants did not show significant differences in fresh root weight or dry root weight, indicating that under aluminum stress conditions, the inhibition of GmAPY1-4 expression had no significant effect on the root weight. This placed additional emphasis on the fact that the silencing of *GmAPY1-4* primarily impacts root elongation without any pronounced corresponding impact on biomass accumulation.

In summary, these findings elucidate the pivotal importance of GmAPY1-4 as a regulator of soybean root growth and aluminum tolerance. Overexpression of this gene not only promotes root elongation but also helps maintain higher root fresh weights and physiological functionality under aluminum stress. In contrast, RNAi interference of GmAPY1-4 hampers root growth and exacerbates growth inhibition under aluminum stress conditions.

## 4. Materials and Methods

### 4.1. Soybean APY Gene Identification

Soybean protein and whole-genome sequences were obtained from the Phytozome v12.1 database (https://phytozome-next.jgi.doe.gov/, accessed on 5 October 2024) [65]. Using the verified adenosine triphosphate diphosphatase (APY) protein sequence from the Arabidopsis Information Resource (TAIR) (https://www.arabidopsis.org/, accessed on 5 October 2024) [66], a BLASTP search was performed against the soybean genome database with a stringent E value threshold of 1.0. Redundant sequences were manually removed. The Plant-mPLoc online tool (http://www.csbio.sjtu.edu.cn/bioinf/plant-multi/, accessed on 26 October 2024) was used to predict the subcellular localization of GmAPY proteins [67], and the Expasy platform (https://web.expasy.org/protparam/, accessed on 28 October 2024) was employed to calculate the molecular weight, isoelectric point, and sequence length [68].

### 4.2. Evolution and Gene Structure Analyses

Homologous APY amino acid sequences were retrieved from soybean (GmAPY), *Arabidopsis thaliana* (AtAPY), *Medicago truncatula* (MtAPY), *Phaseolus vulgaris* (PvAPY), *Zea mays* (ZmAPY), and *Oryza sativa* (OsAPY). A phylogenetic tree was constructed using MEGA 11.0 software, and protein sequences were aligned using ClustalW (https://myhits.sib.swiss/cgi-bin/clustalw, accessed on 20 October 2024) to identify conserved domains. Gene structural data were extracted from genome annotation files, and the conserved motifs were analyzed using MEME 5.5.7 (https://meme-suite.org/meme/tools/meme, accessed on 22 October 2024) [69]. These motifs were visualized using TBtools-II v2.142 [70].

### 4.3. Collinearity Analysis

Collinearity analysis was conducted using the MCScanX program in TBtools-II v2.142 [70]. Genome sequences and annotation files in gff3 format were utilized as input, and default parameters were applied to identify the collinear blocks between soybean and five other species.

### 4.4. Cis-Acting Element Analysis

The 2 kb promoter regions of the *GmAPYs* were analyzed, using the PlantCare website (https://bioinformatics.psb.ugent.be/webtools/plantcare/html/, accessed on 2 November 2024) [71] to predict the cis-acting elements.

### 4.5. GmAPY Expression Analysis

The transcription profiles of *GmAPY* genes in different plant organs (leaves, roots, seeds, pods, and flowers) were analyzed using high-throughput sequencing data from the Phytozome database [65]. The results are presented as a heatmap generated through hierarchical clustering.

### 4.6. qRT-PCR

The soybean seedlings which had grown to the stage when the first trifoliate leaf was fully expanded were transplanted into 50 µM AlCl_3_ solution and treated for 7 days. Subsequently, root samples of the soybeans were collected, and the total RNA was extracted using TransZol Plant reagent (TransGen Biotech, Beijing, China). RNA was treated with DNase I to remove genomic DNA, and cDNA was synthesized using the TransScript^®^ One-Step gDNA Removal and cDNA Synthesis SuperMix (TransGen Biotech, Beijing, China). Then, qRT-PCR analysis was conducted using the PerfectStart™ Green One-Step qPCR SuperMix and specific primers [72]. Gene expression was normalized to the reference gene *GmUKN1* (*Glyma.12g020500*) [73].

### 4.7. Subcellular Localization Analysis

Subcellular localization analysis was performed using 4-week-old *Nicotiana benthamiana* plants. Agrobacterium tumefaciens strain EHA105, containing the CaMV35S-GmAPY1-4-GFP plasmid, was transformed via injection. The OD600 of the Agrobacterium suspension was adjusted to 0.2 with infiltration buffer (10 mM MgCl_2_, 10 mM MES-KOH (pH 5.6), and 150 μM acetosyringone) before injection into tobacco leaves. Fluorescence signals were assessed after 48 h using a Zeiss LSM 700 confocal laser scanning microscope (Zeiss, Oberkochen, Germany).

### 4.8. Soybean Hairy Root Transformation

Hairy root transformation was conducted using *Agrobacterium rhizogenes* strain K599 carrying the plasmids pSoy1-GmAPY1-4-GFP, pSoy1-GFP, pB7GWIWG2-GmAPY1-4-DsRed, and pB7GWIWG2-DsRed [74]. Transgenic roots were screened using qRT-PCR and fluorescence light excitation with an appropriate light source (LUYOR). Positive roots underwent aluminum stress treatment for seven days, and changes in the root length, fresh weight, and dry weight were analyzed.

### 4.9. Statistical Analysis

Statistical analyses were conducted using SPSS 23.0. Experiments were repeated three times, and the results are presented as the mean ± standard deviation (SD). The *p* values were calculated using *t*-tests, with statistical significance being defined as *p* < 0.05. GraphPad Prism 8.0.1 was used to prepare figures.

## 5. Conclusions

In this exhaustive study, the soybean *APY* gene family was thoroughly characterized, and the expression profiles of the included genes were extensively analyzed. A total of 18 *GmAPY* genes were successfully identified and classified into three groups through phylogenetic analysis. Strikingly, those genes within the same subfamily were found to present similar structural features and conserved motif compositions. Additional homology analysis demonstrated an uneven chromosomal distribution of these *GmAPY* genes, with their notable concentration on chromosomes 1, 2, 11, 13, 16, 17, 19, and 20. This observation strongly suggests that segmental duplication has played a pivotal role in the expansion of the *GmAPY* gene family. Cis-regulatory element analysis indicated that *GmAPY* genes are actively involved in hormone regulation and responses to environmental stress. Expression pattern analyses revealed distinct tissue specificity for *GmAPY* genes, along with significant responsivity to aluminum stress. A preliminary functional analysis of the *GmAPY1-4* gene was also conducted, revealing it to localize to the nucleus and be highly expressed in the roots and root nodules, in addition to being positively responsive to aluminum stress in a highly sensitive manner. Further functional characterization showed that overexpression of GmAPY1-4 in soybean hairy roots was sufficient to promote root growth under both normal and aluminum stress conditions, while also preserving higher root fresh weights under aluminum stress. In contrast, the RNA interference of GmAPY1-4 inhibited root growth but without significantly affecting the root dry or fresh weight under either normal or aluminum stress conditions. These findings suggest that GmAPY1-4 plays an essential role in regulating root growth and contributing to plant responses to aluminum stress. Our research on the *GmAPY1-4* gene opens up multiple possibilities for enhancing the genetic aluminum tolerance of soybeans.

## Data Availability

The data presented in this study are available on request from the corresponding author.

## Supplementary Materials

The following supporting information can be downloaded at https://www.mdpi.com/article/10.3390/ijms26051919/s1.

## References

[1] Chiu T.Y., Lao J., Manalansan B., Loqué D., Roux S.J., Heazlewood J.L. (2015). Biochemical characterization of Arabidopsis APYRASE family reveals their roles in regulating endomembrane NDP/NMP homoeostasis. Biochem. J..

[2] Leal D.B., Streher C.A., Neu T.N., Bittencourt F.P., Leal C.A., da Silva J.E., Morsch V.M., Schetinger M.R. (2005). Characterization of NTPDase (NTPDase1; ecto-apyrase; ecto-diphosphohydrolase; CD39; EC 3.6.1.5) activity in human lymphocytes. Biochim. Biophys. Acta.

[3] Knowles A.F. (2011). The GDA1_CD39 superfamily: NTPDases with diverse functions. Purinergic Signal..

[4] Farani P.S.G., Marconato D.G., Emidio N.B., Pereira V.R.D., Alves Junior I.J., da Silveira L.S., Couri M.R.C., Vasconcelos E.G., Castro-Borges W., Filho A.A.S. (2020). Screening of plant derived chalcones on the inhibition of potato apyrase: Potential protein biotechnological applications in health. Int. J. Biol. Macromol..

[5] Handa M., Guidotti G. (1996). Purification and cloning of a soluble ATP-diphosphohydrolase (apyrase) from potato tubers (*Solanum tuberosum*). Biochem. Biophys. Res. Commun..

[6] Chiu T.Y., Christiansen K., Moreno I., Lao J., Loqué D., Orellana A., Heazlewood J.L., Clark G., Roux S.J. (2012). AtAPY1 and AtAPY2 function as Golgi-localized nucleoside diphosphatases in Arabidopsis thaliana. Plant Cell Physiol..

[7] Liu W., Ni J., Shah F.A., Ye K., Hu H., Wang Q., Wang D., Yao Y., Huang S., Hou J. (2019). Genome-wide identification, characterization and expression pattern analysis of APYRASE family members in response to abiotic and biotic stresses in wheat. PeerJ.

[8] Chowdhury A.T., Hasan M.N., Bhuiyan F.H., Islam M.Q., Nayon M.R.W., Rahaman M.M., Hoque H., Jewel N.A., Ashrafuzzaman M., Prodhan S.H. (2023). Identification, characterization of Apyrase (APY) gene family in rice (*Oryza sativa*) and analysis of the expression pattern under various stress conditions. PLoS ONE.

[9] Cohn J.R., Uhm T., Ramu S., Nam Y.W., Kim D.J., Penmetsa R.V., Wood T.C., Denny R.L., Young N.D., Cook D.R. (2001). Differential regulation of a family of apyrase genes from Medicago truncatula. Plant Physiol..

[10] Takahashi H., Toyoda K., Hirakawa Y., Morishita K., Kato T., Inagaki Y., Ichinose Y., Shiraishi T. (2006). Localization and responsiveness of a cowpea apyrase VsNTPase1 to phytopathogenic microorganisms. J. Gen. Plant Pathol..

[11] Riewe D., Grosman L., Fernie A.R., Wucke C., Geigenberger P. (2008). The potato-specific apyrase is apoplastically localized and has influence on gene expression, growth, and development. Plant Physiol..

[12] Riku O., Takeshi T., Nishimura N., Shogo U., Takahide T., Nobuyuki K. (2011). Purification and Biochemical Characterization of a Novel Ecto-Apyrase, MP67, from Mimosa pudica. Plant Physiol..

[13] Murphy A., Chen S., Zhao R., Lin S., Deng S., Zhao N., Zhang Y., Zhang Y., Hou S., Liu J. (2020). Populus euphratica WRKY1 binds the promoter of H+-ATPase gene to enhance gene expression and salt tolerance. J. Exp. Bot..

[14] Wolf C., Hennig M., Romanovicz D., Steinebrunner I. (2007). Developmental defects and seedling lethality in apyrase AtAPY1 and AtAPY2 double knockout mutants. Plant Mol. Biol..

[15] Steinebrunner I., Wu J., Sun Y., Corbett A., Roux S.J. (2003). Disruption of apyrases inhibits pollen germination in Arabidopsis. Plant Physiol..

[16] Wu J., Steinebrunner I., Sun Y., Butterfield T., Torres J., Arnold D., Gonzalez A., Jacob F., Reichler S., Roux S.J. (2007). Apyrases (nucleoside triphosphate-diphosphohydrolases) play a key role in growth control in Arabidopsis. Plant Physiol..

[17] Gupta S., Guérin A., Herger A., Hou X., Schaufelberger M., Roulard R., Diet A., Roffler S., Lefebvre V., Wicker T. (2024). Growth-inhibiting effects of the unconventional plant APYRASE 7 of Arabidopsis thaliana influences the LRX/RALF/FER growth regulatory module. PLOS Genet..

[18] Sabharwal T., Lu Z., Slocum R.D., Kang S., Wang H., Jiang H.W., Veerappa R., Romanovicz D., Nam J.C., Birk S. (2022). Constitutive expression of a pea apyrase, psNTP9, increases seed yield in field-grown soybean. Sci. Rep..

[19] Zhang Z., Yao J., Jiang Z., Huang X., Wang S., Xu F. (2024). Golgi-localized APYRASE 1 is critical for Arabidopsis growth by affecting cell wall integrity under boron deficiency. Physiol. Plant..

[20] Clark G., Fraley D., Steinebrunner I., Cervantes A., Onyirimba J., Liu A., Torres J., Tang W., Kim J., Roux S.J. (2011). Extracellular nucleotides and apyrases regulate stomatal aperture in Arabidopsis. Plant Physiol..

[21] Jeter C.R., Tang W., Henaff E., Butterfield T., Roux S.J. (2004). Evidence of a novel cell signaling role for extracellular adenosine triphosphates and diphosphates in Arabidopsis. Plant Cell.

[22] Lim M.H., Wu J., Yao J., Gallardo I.F., Dugger J.W., Webb L.J., Huang J., Salmi M.L., Song J., Clark G. (2014). Apyrase Suppression Raises Extracellular ATP Levels and Induces Gene Expression and Cell Wall Changes Characteristic of Stress Responses. Plant Physiol..

[23] Deng S., Sun J., Zhao R., Ding M., Zhang Y., Sun Y., Wang W., Tan Y., Liu D., Ma X. (2015). Populus euphratica APYRASE2 Enhances Cold Tolerance by Modulating Vesicular Trafficking and Extracellular ATP in Arabidopsis Plants. Plant Physiol..

[24] Zhang Y., Sun Y., Liu X., Deng J., Yao J., Zhang Y., Deng S., Zhang H., Zhao N., Li J. (2021). Populus euphratica Apyrases Increase Drought Tolerance by Modulating Stomatal Aperture in Arabidopsis. Int. J. Mol. Sci..

[25] Ma C., Ma S., Yu Y., Feng H., Wang Y., Liu C., He S., Yang M., Chen Q., Xin D. (2024). Transcriptome-wide m 6 A methylation profiling identifies GmAMT1;1 as a promoter of lead and cadmium tolerance in soybean nodules. J. Hazard. Mater..

[26] Ma C., Wang J., Gao Y., Dong X., Feng H., Yang M., Yu Y., Liu C., Wu X., Qi Z. (2024). The type III effector NopL interacts with GmREM1a and GmNFR5 to promote symbiosis in soybean. Nat. Commun..

[27] Pang Z., Yin W., Wang Y., Zeng W., Peng H., Liang Y. (2023). Silicon-phosphorus pathway mitigates heavy metal stress by buffering rhizosphere acidification. Sci. Total Environ..

[28] Ur Rahman S., Han J.C., Ahmad M., Ashraf M.N., Khaliq M.A., Yousaf M., Wang Y., Yasin G., Nawaz M.F., Khan K.A. (2024). Aluminum phytotoxicity in acidic environments: A comprehensive review of plant tolerance and adaptation strategies. Ecotoxicol. Environ. Saf..

[29] Waadt R., Seller C.A., Hsu P.K., Takahashi Y., Munemasa S., Schroeder J.I. (2022). Plant hormone regulation of abiotic stress responses. Nat. Rev. Mol. Cell Biol..

[30] GB C., RO M., MP F., ML S., SJ R. (2014). Breakthroughs spotlighting roles for extracellular nucleotides and apyrases in stress responses and growth and development. Plant Sci..

[31] Tanaka K., Gilroy S., Jones A.M., Stacey G. (2010). Extracellular ATP signaling in plants. Trends Cell Biol..

[32] Clark G., Roux S.J. (2018). Role of Ca 2+ in Mediating Plant Responses to Extracellular ATP and ADP. Int. J. Mol. Sci..

[33] Demidchik V., Shang Z., Shin R., Thompson E., Rubio L., Laohavisit A., Mortimer J.C., Chivasa S., Slabas A.R., Glover B.J. (2009). Plant extracellular ATP signalling by plasma membrane NADPH oxidase and Ca2+ channels. Plant J..

[34] Sharif Y., Mamadou G., Yang Q., Cai T., Zhuang Y., Chen K., Deng Y., Khan S.A., Ali N., Zhang C. (2023). Genome-Wide Investigation of Apyrase (APY) Genes in Peanut (*Arachis hypogaea* L.) and Functional Characterization of a Pod-Abundant Expression Promoter AhAPY2-1p. Int. J. Mol. Sci..

[35] Navarro-Gochicoa M.T., Sylvie C., Andréas N., Julie V.C. (2003). Expression of the Apyrase-Like APY1 Genes in Roots ofMedicago truncatula Is Induced Rapidly and Transiently by Stress and Not by Sinorhizobium meliloti or Nod Factors. Plant Physiol..

[36] He Z., Zhang J., Jia H., Zhang S., Sun X., Nishawy E., Zhang H., Dai M. (2024). Genome-wide identification and analyses of ZmAPY genes reveal their roles involved in maize development and abiotic stress responses. Mol. Breed..

[37] Liu L., Xu L., Jia Q., Pan R., Oelmüller R., Zhang W., Wu C. (2019). Arms race: Diverse effector proteins with conserved motifs. Plant Signal. Behav..

[38] Perry J., Ashworth A. (1999). Evolutionary rate of a gene affected by chromosomal position. Curr. Biol..

[39] Rengong M., Ling Z., Yongqing Y., Longfu Z., Ze-Hao H., Ling J., Wang B.C. (2019). Apyrases in Arabidopsis thaliana. Biol. Plant..

[40] Goldammer G., Neumann A., Strauch M., Müller-McNicoll M., Heyd F., Preußner M. (2018). Characterization of cis-acting elements that control oscillating alternative splicing. RNA Biol..

[41] Hauser F., Li Z., Waadt R., Schroeder J.I. (2017). SnapShot: Abscisic Acid Signaling. Cell.

[42] Binenbaum J., Weinstain R., Shani E. (2018). Gibberellin Localization and Transport in Plants. Trends Plant Sci..

[43] Cohen J.D., Strader L.C. (2024). An auxin research odyssey: 1989-2023. Plant Cell.

[44] Peng Y., Yang J., Li X., Zhang Y. (2021). Salicylic Acid: Biosynthesis and Signaling. Annu. Rev. Plant Biol..

[45] Yu X., Zhang W., Zhang Y., Zhang X., Lang D., Zhang X. (2019). The roles of methyl jasmonate to stress in plants. Funct. Plant Biol..

[46] Liu X., Wu J., Clark G., Lundy S., Lim M., Arnold D., Chan J., Tang W., Muday G.K., Gardner G. (2012). Role for apyrases in polar auxin transport in Arabidopsis. Plant Physiol..

[47] Hao L.H., Wang W.X., Chen C., Wang Y.F., Liu T., Li X., Shang Z.L. (2012). Extracellular ATP promotes stomatal opening of Arabidopsis thaliana through heterotrimeric G protein α subunit and reactive oxygen species. Mol. Plant.

[48] Veerappa R., Slocum R.D., Siegenthaler A., Wang J., Clark G., Roux S.J. (2019). Ectopic expression of a pea apyrase enhances root system architecture and drought survival in Arabidopsis and soybean. Plant Cell Environ..

[49] Ma Y., Zhang Y., Xu J., Qi J., Liu X., Guo L., Zhang H. (2024). Research on the Mechanisms of Phytohormone Signaling in Regulating Root Development. Plants.

[50] Wu J., Gao F., Li T., Guo H., Zhang L., Fan Y., Chen A., Wang J., Shi F., Shan G. (2021). Genome-Wide cis -Regulatory Element Based Discovery of Auxin-Responsive Genes in Higher Plant. Genes.

[51] Falquetto-Gomes P., Silva W.J., Siqueira J.A., Araújo W.L., Nunes-Nesi A. (2024). From epidermal cells to functional pores: Understanding stomatal development. J. Plant Physiol..

[52] Liu N., You J., Shi W., Liu W., Yang Z. (2011). Salicylic acid involved in the process of aluminum induced citrate exudation in *Glycine max* L.. Plant Soil..

[53] Hou N., You J., Pang J., Xu M., Chen G., Yang Z. (2009). The accumulation and transport of abscisic acid in soybean (*Glycine max* L.) under aluminum stress. Plant Soil..

[54] Sun P., Tian Q.Y., Chen J., Zhang W.H. (2010). Aluminium-induced inhibition of root elongation in Arabidopsis is mediated by ethylene and auxin. J. Exp. Bot..

[55] Wang H., Wang H., Liu H., Wan T., Li Y., Zhang K., Shabala S., Li X., Chen Y., Yu M. (2024). Aluminium stress-induced modulation of root gravitropism in pea (*Pisum sativum*) via auxin signalling. Plant Physiol. Biochem..

[56] Yamamoto Y., Kobayashi Y., Devi S.R., Rikiishi S., Matsumoto H. (2002). Aluminum toxicity is associated with mitochondrial dysfunction and the production of reactive oxygen species in plant cells. Plant Physiol..

[57] Exley C. (2004). The pro-oxidant activity of aluminum. Free Radic. Biol. Med..

[58] Sharma P., Dubey R.S. (2007). Involvement of oxidative stress and role of antioxidative defense system in growing rice seedlings exposed to toxic concentrations of aluminum. Plant Cell Rep..

[59] Cíntia Oliveira S., Danielle Santos B., Roberto N.-S., da Silva A.A., Vanessa do Rosário R., Michel Filiphy Silva S., Allan de Marcos L., Maximiller D.-B., Cléberson R. (2023). Modulation of the antioxidant system and primary metabolism confers aluminum stress tolerance in soybean. Acta Physiol. Plant..

[60] Wang Z., Lun L., Hui S., Guo L., Zhang J., Li Y., Xu J., Zhang X., Guo Y., Zhang N. (2020). Jasmonate and aluminum crosstalk in tomato: Identification and expression analysis of WRKYs and ALMTs during JA/Al-regulated root growth. Plant Physiol. Biochem..

[61] Ali M.S., Baek K.H. (2020). Jasmonic Acid Signaling Pathway in Response to Abiotic Stresses in Plants. Int. J. Mol. Sci..

[62] Choudhury S., Sharma P. (2014). Aluminum stress inhibits root growth and alters physiological and metabolic responses in chickpea (*Cicer arietinum* L.). Plant Physiol. Biochem..

[63] Riaz M., Yan L., Wu X., Hussain S., Aziz O., Jiang C. (2018). Mechanisms of organic acids and boron induced tolerance of aluminum toxicity: A review. Ecotoxicol. Environ. Saf..

[64] Pan X., Dong G., He X., Wang R., Xu R., Mu T. (2020). Effects of Al stress on the growth and nitrogen uptake of maize varieties with different Al tolerance as related with Al chemical forms on root surfaces. J. Soils Sediments.

[65] Goodstein D.M., Shu S., Howson R., Neupane R., Hayes R.D., Fazo J., Mitros T., Dirks W., Hellsten U., Putnam N. (2012). Phytozome: A comparative platform for green plant genomics. Nucleic Acids Res..

[66] Lamesch P., Berardini T.Z., Li D., Swarbreck D., Wilks C., Sasidharan R., Muller R., Dreher K., Alexander D.L., Garcia-Hernandez M. (2012). The Arabidopsis Information Resource (TAIR): Improved gene annotation and new tools. Nucleic Acids Res..

[67] Chou K.C., Shen H.B. (2010). Plant-mPLoc: A top-down strategy to augment the power for predicting plant protein subcellular localization. PLoS ONE.

[68] Wilkins M.R., Gasteiger E., Bairoch A., Sanchez J.C., Williams K.L., Appel R.D., Hochstrasser D.F. (1999). Protein identification and analysis tools in the ExPASy server. Methods Mol. Biol..

[69] Bailey T.L., Boden M., Buske F.A., Frith M., Grant C.E., Clementi L., Ren J., Li W.W., Noble W.S. (2009). MEME SUITE: Tools for motif discovery and searching. Nucleic Acids Res..

[70] Chen C., Wu Y., Li J., Wang X., Zeng Z., Xu J., Liu Y., Feng J., Chen H., He Y. (2023). TBtools-II: A “one for all, all for one” bioinformatics platform for biological big-data mining. Mol. Plant.

[71] Lescot M., Déhais P., Thijs G., Marchal K., Moreau Y., Van de Peer Y., Rouzé P., Rombauts S. (2002). PlantCARE, a database of plant cis-acting regulatory elements and a portal to tools for in silico analysis of promoter sequences. Nucleic Acids Res..

[72] Wang J., Ma C., Ma S., Zheng H., Tian H., Wang X., Wang Y., Jiang H., Wang J., Zhang Z. (2023). Genetic variation in GmCRP contributes to nodulation in soybean (*Glycine max* Merr.). Crop J..

[73] Li Q., Fan C.M., Zhang X.M., Fu Y.F. (2012). Validation of reference genes for real-time quantitative PCR normalization in soybean developmental and germinating seeds. Plant Cell Rep..

[74] Katalin T., Josef B., Gary S. (2016). Generation of Soybean (*Glycine max*) Transient Transgenic Roots. Curr. Protoc. Plant Biol..

