# Supplementary material for: Genome-Wide Analysis of Soybean Apyrase Gene Family and Functional Characterization of GmAPY1-4 Responses to Aluminum Stress
Source: Int J Mol Sci. 2025 Feb 23;26(5):1919. doi: 10.3390/ijms26051919 (PMC11900418; doi:10.3390/ijms26051919)
Supplement: Supplementary file 1 [file ijms-26-01919-s001.zip › SupplementaryFigures/SupplementaryFigures information.pdf]

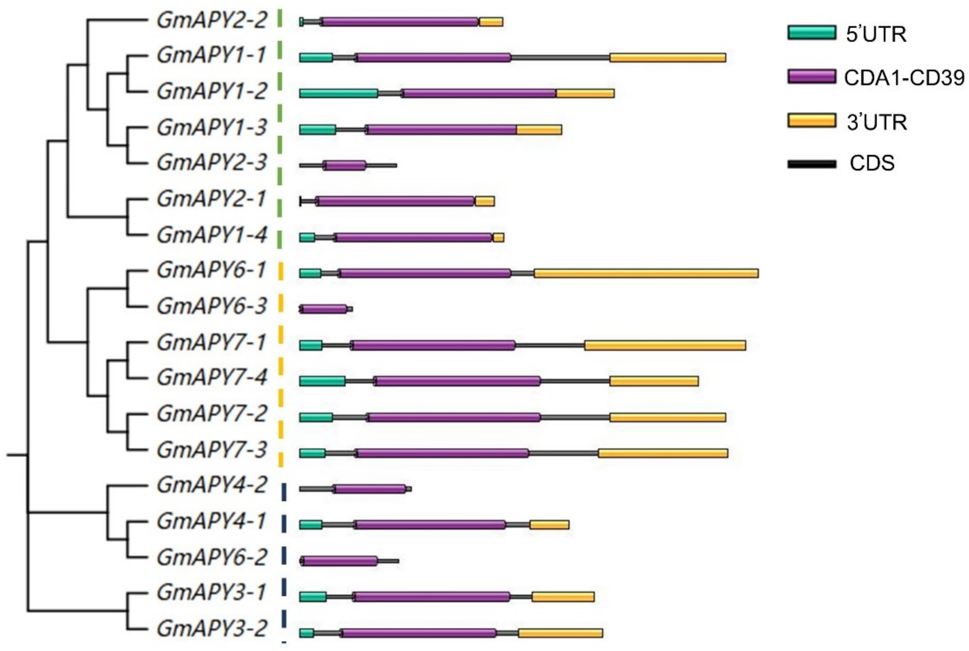

Figure S1. The conserved ACR domain of GmAPYs.

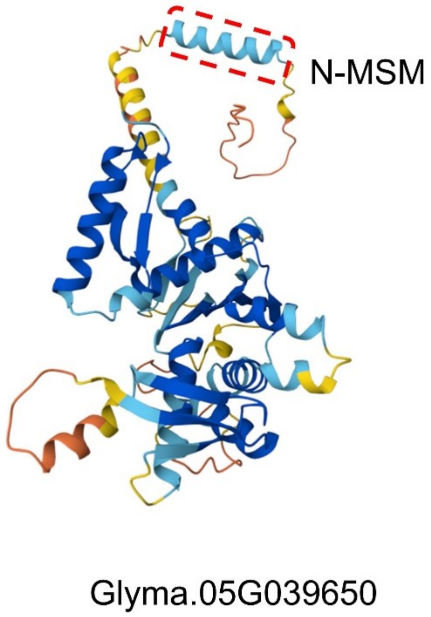

Figure S2. 3D structure analysis of *Glyma.05G039650*. Red box: the N-terminal membrane spanning motif (MSM).

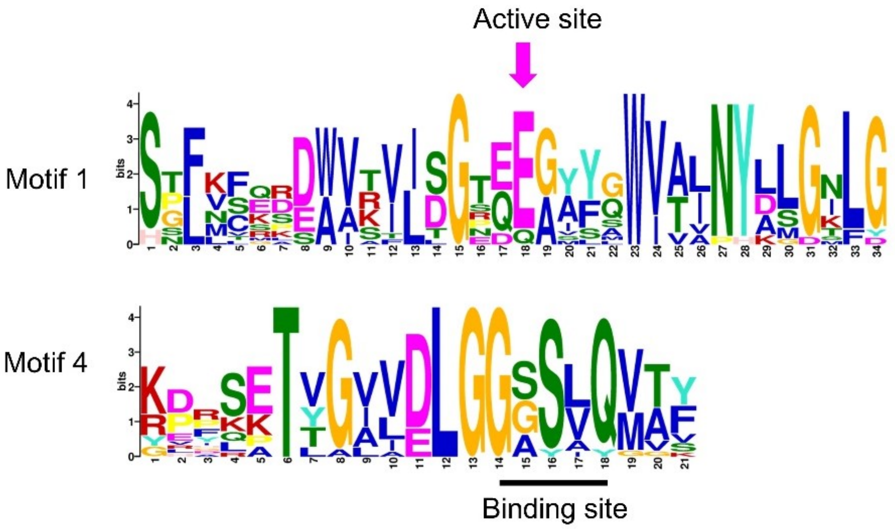

Figure S3. The patterns of Motifs 1 and Motifs 4. Active site: Proton acceptor. Binding site: ATP.
