# Supplementary figures and images for: Genome-Wide Analysis of Soybean Apyrase Gene Family and Functional Characterization of GmAPY1-4 Responses to Aluminum Stress
Source: Int J Mol Sci. 2025 Feb 23;26(5):1919. doi: 10.3390/ijms26051919 (PMC11900418; doi:10.3390/ijms26051919)

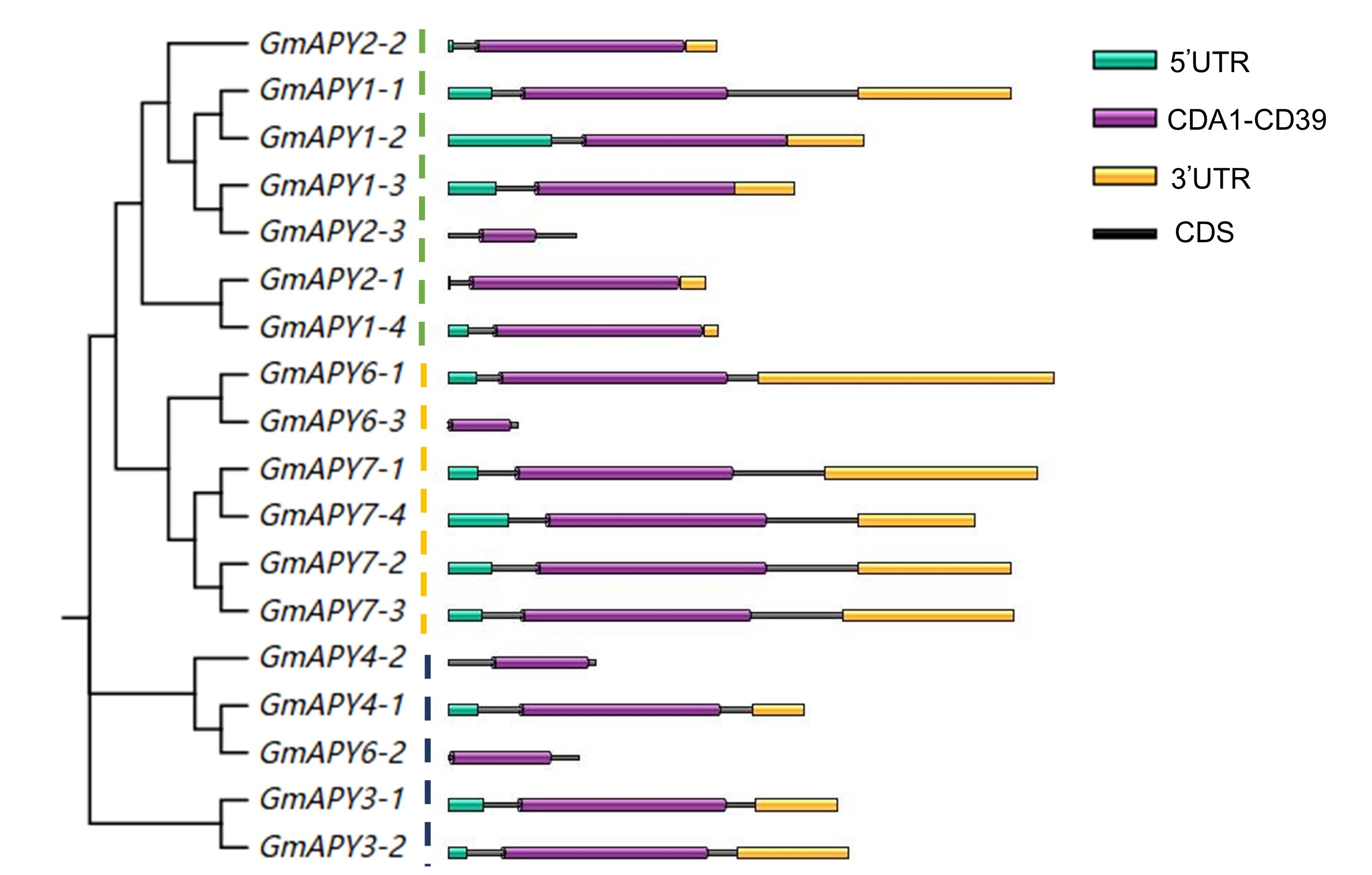

Supplement: Supplementary file 1 [file ijms-26-01919-s001.zip › SupplementaryFigures/SupplementaryFigure 1.jpg]

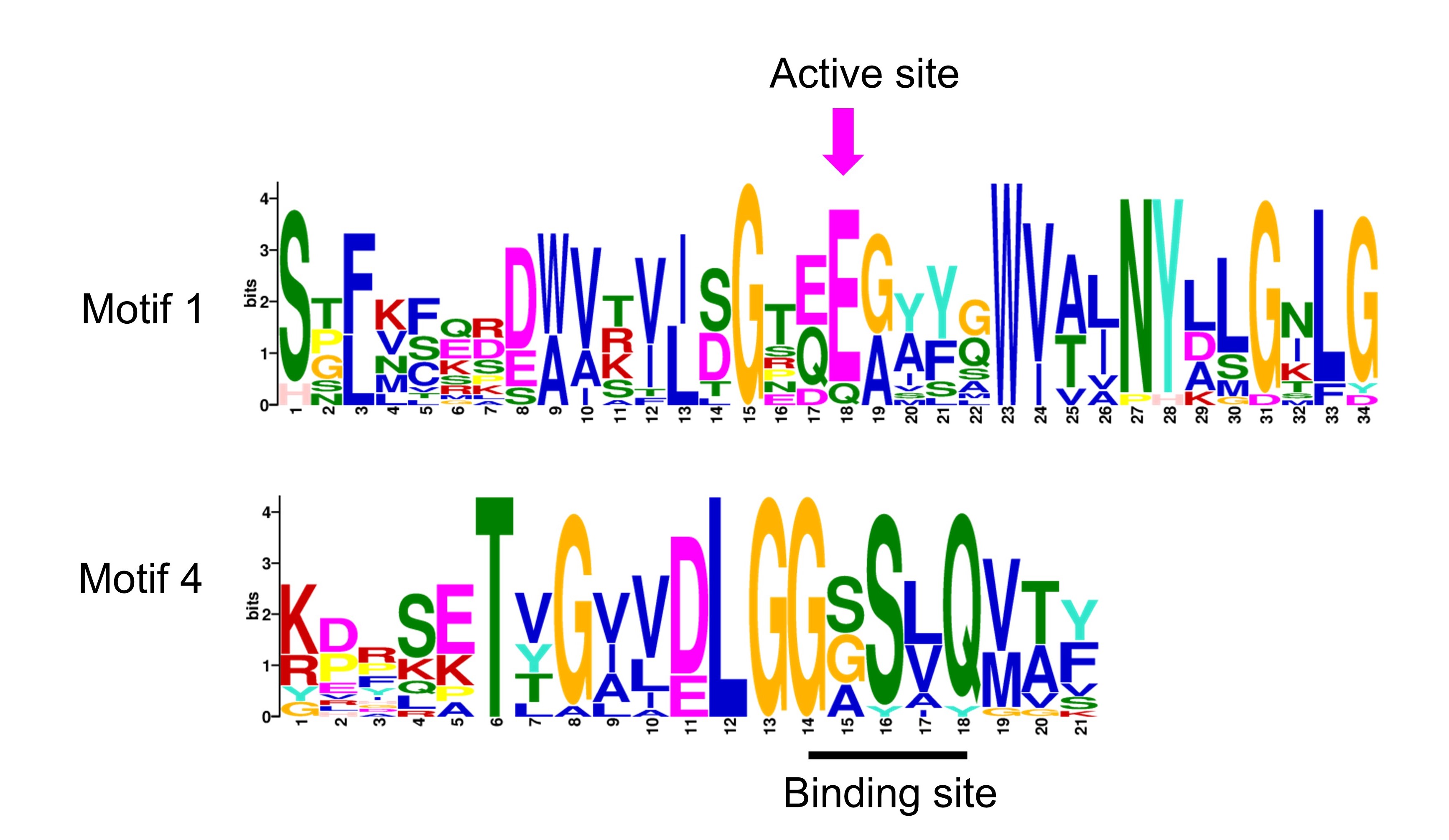

Supplement: Supplementary file 1 [file ijms-26-01919-s001.zip › SupplementaryFigures/SupplementaryFigure 3.jpg]

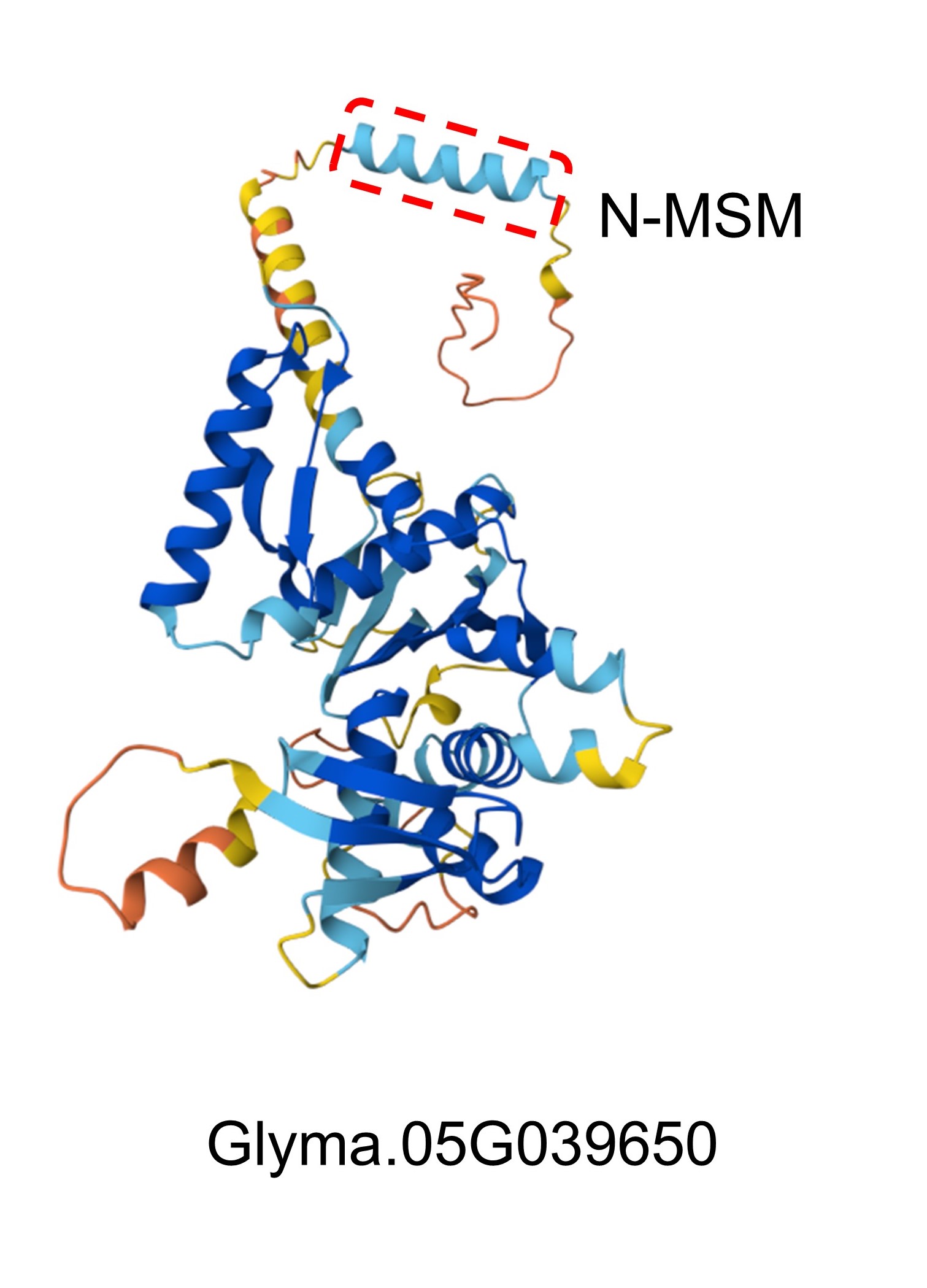

Supplement: Supplementary file 1 [file ijms-26-01919-s001.zip › SupplementaryFigures/SupplementaryFigures 2.jpg]
